# HSBDF-Derived Bioactive Components Broadly Inhibit Enteroviruses by Targeting 3C Protease and Attenuating Inflammatory Responses

**DOI:** 10.3390/biology14111615

**Published:** 2025-11-18

**Authors:** Ruolan Hu, Lin Guan, Siyue Li, Chunlin Liu, Gang Huang, Fuxing Lou, Hongzheng Jiang, Shuqi Wang, Zehan Pang, Yaxin Wang, Zhenlu Li, Han Zhang, Yigang Tong, Huahao Fan, Bixia Hong

**Affiliations:** 1 School of Life Sciences, Tianjin University, Tianjin 300072, China; 2 College of Life Science and Technology, Beijing University of Chemical Technology, Beijing 100029, Chinajianghz@huataiyc.com (H.J.);; 3 State Key Laboratory of Component-Based Chinese Medicine, Tianjin University of Traditional Chinese Medicine, Tianjin 301617, China; 4 State Key Laboratory of Synthetic Biology, Tianjin University, Tianjin 300072, China; 5 State Key Laboratory of Pathogen and Biosecurity, Beijing Institute of Microbiology and Epidemiology, Beijing 100071, China; 6 State Key Laboratory of Respiratory Disease, School of Basic Medical Science, Guangzhou Medical University, Guangzhou 511436, China

**Keywords:** enterovirus, Huashi Baidu Formula (HSBDF), flavonoids, 3C protease inhibitor, anti-inflammatory

## Abstract

Human enteroviruses are important pathogens of hand-foot-and-mouth disease, poliomyelitis, and encephalitis, etc., posing substantial global health burdens with no specific approved therapeutics. While traditional Chinese medicine (TCM) has demonstrated antiviral potential during the COVID-19 pandemic, its efficacy and pharmacodynamic material basis against enteroviruses remains underexplored. Here, we systematically characterized the broad-spectrum anti-enterovirus activity of Huashi Baidu Formula (HSBDF), a clinically approved TCM for COVID-19, and identified three flavonoid compounds as its active components responsible for this antiviral effect. Transcriptomics analysis revealed that HSBDF attenuated CV-A9-induced inflammation by modulating MAPK and NF-κB signaling pathways. High Performance Liquid Chromatography-Tandem Mass Spectrometry (HPLC-MS/MS) analysis identified 152 chemical compounds in HSBDF, among which three flavonoids—velutin, isorhamnetin, and (−)-epicatechin gallate—exhibited potent pan-enteroviral inhibition. Mechanistically, these compounds suppressed the activity of 3C proteases in enteroviruses, while concurrently attenuating CV-A9-induced upregulation of IL-6, TNF-α, MCP-1, and COX-2. Utilizing a BALB/c young mouse model, it was demonstrated that the HSBDF and its compound velutin effectively suppressed viral replication in vivo. Collectively, this study advances TCM-based strategies for enterovirus therapy exemplified by HSBDF and highlights flavonoid scaffolds as promising candidates for developing broad-spectrum anti-enteroviral agents.

## Simple Summary

Human enteroviruses are important pathogens of hand-foot-and-mouth disease, poliomyelitis, and encephalitis, etc., and there are currently no medicines that specifically treat them. Although traditional Chinese herbal medicines showed some antiviral activity during the COVID-19 pandemic, their efficacy and pharmacodynamic material basis against enteroviruses remains underexplored. In this work, we characterized a Chinese herbal mixture, Huashi Baidu Formula, which has already been approved for COVID-19, and found that it shows broad-spectrum anti-enterovirus activity. We identified 152 chemical compounds in Huashi Baidu Formula, among which three flavonoids—velutin, isorhamnetin, and (−)-epicatechin gallate—exhibited potent pan-enteroviral inhibition activity. These compounds block a key viral enzyme and also calm the body’s inflammatory response. Tests in young mice showed that the herbal formula and the compound velutin greatly reduced viral growth. The study demonstrates that this herbal formula and its flavonoid components could become safe, affordable treatments for enterovirus infections, helping to lower the global health burden of these diseases.

## 1. Introduction

Enteroviruses are a group of single-stranded, positive-sense RNA viruses with no envelop, comprising over 280 serotypes [1], including enterovirus A71 (EV-A71), coxsackievirus A16 (CV-A16), echoviruses, poliovirus, etc. These pathogens are associated with diverse clinical manifestations such as hand-foot-and-mouth disease (HFMD), meningitis, and acute flaccid myelitis [2,3,4,5]. Despite the rollout of polio vaccines significantly reducing poliovirus infections, poliovirus remains endemic in certain regions [6], while non-polio enteroviruses continue to escalate in prevalence. Notably, EV-A71 has triggered recurrent HFMD epidemics in the Asia-Pacific region, resulting in tens of thousands of severe cases and fatalities [7]. Concurrently, EV-D68 has been linked to severe respiratory illnesses and neurological paralysis in children across Europe and North America, underscoring its transregional transmission threats [8]. These viruses disproportionately affect infants, young children, and immunocompromised individuals, with some infections causing irreversible neurological sequelae or lifelong disabilities, imposing a substantial socioeconomic burden [9]. However, no approved targeted antiviral therapies exist for enterovirus infections [9], and only four serotype-specific vaccines have been licensed, which fail to confer heterotypic cross-protection against other circulating strains. Therefore, effective broad-spectrum anti-enteroviral inhibitors remain urgently needed to address public health challenges posed by the co-prevalence of multiple serotypes.

Traditional Chinese medicine (TCM) formulations demonstrate multi-component and multi-target therapeutic advantages in combating major infectious diseases, as evidenced by their critical roles in COVID-19 containment and artemisinin-based malaria treatment. However, for HFMD—a prevalent pediatric enterovirus infection—while symptom alleviation (e.g., fever and mucosal lesions) has been achieved by formulations like Ganlu Xiaodu Dan and Qingwen Baidu Yin [10], there exist significant gaps in standardized efficacy evaluation systems and mechanistic investigations. Current clinical management of HFMD relies on ribavirin-based antiviral regimens; however, the emergence of drug-resistant viral strains poses a major therapeutic challenge [11,12]. Therefore, developing TCM formulations integrating both antiviral activity and immunomodulatory functions represents an urgent therapeutic imperative.

Huashi Baidu Formula (HSBDF), a COVID-19 therapeutic agent approved by China’s National Medical Products Administration (NMPA), comprises 14 medicinal components including Ephedrae Herba (Mahuang), Pogostemonis Herba (Guanghuoxiang), and Gypsum Fibrosum (Shiga), etc. [13,14,15]. Its granule formulation is commercially available for managing epidemic diseases associated with dampness-toxin invasion of the lung syndrome in traditional Chinese medicine (TCM) theory. Clinical trials have revealed that HSBDF has better effects on SARS-CoV-2 RNA clearance and reducing inflammation [16,17,18]. Notably, severe HFMD cases exhibit pathophysiological parallels with acute COVID-19, characterized by sustained pyrexia, respiratory tract inflammation, and systemic immune hyperactivation. These shared immunopathological mechanisms suggest HSBDF’s potential cross-application against enteroviral infections via conserved regulatory networks. To evaluate its anti-HFMD potential, we implemented an integrative pharmacology strategy combining: in vitro antiviral assessments, High Performance Liquid Chromatography-Tandem Mass Spectrometry (HPLC-MS/MS)-based phytochemical profiling, transcriptomic analysis, and molecular docking. This systematic approach identified velutin, isorhamnetin, and (−)-epicatechin gallate as key constituents exhibiting multimodal mechanisms (antiviral and anti-inflammatory effects), providing a mechanistic framework for repurposing HSBDF against enterovirus diseases while advancing natural antiviral drug discovery.

## 2. Materials and Methods

### 2.1. Cell Lines and Viruses

Human rhabdomyosarcoma (RD) cells, human hepatocellular carcinoma (Huh-7) cells, and Vero E6 cells were obtained from the American Type Culture Collection (ATCC). These cells were cultured in Dulbecco’s Modified Eagle’s Medium (Gibco, Grand Island, NY, USA) with 10% fetal bovine serum (FBS; PAN, Aidenbach, Germany) and 1% antibiotic-antimycotic solution (Gibco, NY, USA) at 37 °C with 5% CO_2_.

The CV-A9 strain BUCT01 (GenBank accession No. MW192795) was isolated from the feces of a pediatric patient with HFMD in China with high-throughput sequencing. EV-A71 Wenzhou strain, CV-B3 (GenBank accession No. JX312064.1), poliovirus 1 (PV1) (vaccine strain), and Echo-11 were kindly gifted by Dr. Zhen Luo, Jinan University, China. CV-A9, EV-A71, PV1, and Echo-11 were propagated in RD cells and CV-B3 was propagated in Vero E6 cells. All the viruses were titered by plaque assay.

### 2.2. HSBDF and Compounds

HSBDF was a finished pharmaceutical product made by Efong Pharmaceuticral (Foshan, Guangdong, China). The required mass of HSBDF was measured based on the experimental concentration, dissolved in 65 °C water, and subsequently filtered using a 0.22 μm membrane. Identification of small molecule compounds in the formula was performed by HPLC-MS/MS at Sci-Tech Innovation (Qingdao, China). The 119 compounds tested in this article were all commercially available in the components. See Tables S1 and S2 for details.

### 2.3. EC_50_ and CC_50_ Evaluations

The EC_50_ and CC_50_ evaluations were conducted in accordance with previously outlined methodologies and calculated through nonlinear regression analysis [19]. The MOI of virus infection was 0.001 (CV-A9 and CV-B3), 0.1 (EV-A71), 0.003 (PV1), and 0.005 (Echo-11), respectively.

### 2.4. Plaque Assay

The plaque assays on RD cells or Vero E6 cells were conducted in accordance with established protocols to assess the production of infectious virions [20].

### 2.5. Time-of-Addition Assay

The experiment was performed as previously described [21]. Drugs were added at different stages in the life cycle of viral infection. At 14 h.p.i., cells and supernatant from the treatment and control groups were collected for RT-qPCR analysis or plaque assay. The sequences of relevant RT-qPCR primers are listed in Table S3. The MOI of virus infection was the same as in the experiments in EC_50_ evaluations.

### 2.6. Virus Attachment and Internalization Assay

RD cells were cultured in 48-well plates, and the treatments were conducted at 90–100% cell density. The experimental programs were performed as described previously [22]. The MOI of EV-A71 and CV-A9 was 5.

### 2.7. Determination of the Post-Entry Inhibitory Effect

The experiments were performed as previously described [23]. RD cells were cultured in 48-well plates and infected with the virus for 2 h. Then they were treated with drug-containing media. Cycloheximide (CHX), as a positive control, has been shown to inhibit enterovirus genome replication [24]. Subsequently, cell lysates and supernatants were harvested at various time intervals for RT-qPCR analysis to assess viral expression levels. The MOI of virus infection was the same as in the experiments in EC_50_ evaluations.

### 2.8. RNA-Sequencing (RNA-Seq) Processing and Analysis

Four experimental groups were set up. In the drug-treated group, RD cells were infected with CV-A9 (MOI = 0.001) and treated with 1 mg/mL HSBDF. Additionally, several control groups were established, including a drug-only group in which RD cells were treated with 1 mg/mL HSBDF, a virus-only infection group in which RD cells were infected with CV-A9, and a blank control group consisting solely of RD cells. RNA was collected after 36 h of culture and sequenced using the standard Illumina protocol (Annoroad Gene Technology, Beijing, China). Reads were mapped to the genome using HISAT2 v2.1.1. Each gene was counted using the HTSeq-count 0.9.1. R 4.3.0 and Rtools were used to identify the differentially expressed genes (DEGs) with a fold change of ≥2 and an adjusted *p*-value of <0.05. Raw data can be found in Tables S4 and S5. Gene ontology analysis was performed with a web-based tool, Metascape (https://metascape.org), with differentially expressed genes obtained as described above. A pathway with a *p*-value < 0.05 was considered as a significantly enriched pathway. The interaction network for each significantly enriched pathway and the protein–protein interaction (PPI) network was drawn by Cytoscape 3.9.1, which is also included on the Metascape website. The heatmap analysis was performed by using heatmap tools in Hiplot Pro (https://hiplot.com.cn/), a comprehensive web service for biomedical data analysis and visualization.

### 2.9. Primary Screening for Antiviral Compounds of HSBDF

The compounds of HSBDF were determined by mass spectrometry at Sci-Tech Innovation (Qingdao, China), and a total of 119 molecules in this formula were purchased from TargetMol (Boston, MA, USA) for the antiviral drug screening. The RD cells in 96-well plates were co-treated with viruses and compounds (10 μM). At 36 h.p.i., the potential antiviral compounds were determined through the cytopathic effects (CPEs).

### 2.10. Fluorescence Resonance Energy Transfer (FRET)-Based Protease Assays

The 2A and 3C proteases of CV-A9 (GenBank: MW192795) which were required in the experiment were expressed in *E. coli* BL21(DE3) using pET28a-2A or pET28a-3C recombinant plasmids constructed in our laboratory. The 3C protease of EV-A71 (GenBank: KP861243.1) was also expressed by a recombinant plasmid constructed by us using the same method. Additionally, a recombinant plasmid for CV-B3 (GenBank: JX312064.1c) 3C protease was provided by Beijing Xianghong Biotechnology Co., Ltd. (Beijing, China).

Based on the cleavage activities of enterovirus 2A and 3C proteases, the fluorescence enhancement due to corresponding peptide cleavage was monitored at 538 nm with excitation at 355 nm by a Synergy H1 microplate reader (BioTek, Norcross, GA, USA). Kinetic measurements for the proteases were performed in a 50 mM MES buffer (pH 6.5) containing different concentrations of fluorescent peptides (FRET-1: Dabcyl-KTSAVLQSGFRKME-Edans for CV-A9 and CV-B3 3C^pro^; FRET-2: Dabcyl-KNTHGAFGHQSGAE-Edans for CV-A9 2A^pro^; FRET-3: Dabcyl-RTATVQGPSLDFE-Edans for EV-A71 3C^pro^). To measure the inhibitory effects of the drugs on protease activity, each drug was separately pretreated with the enzyme for 30 min, and the cleavage of the substrate peptide was monitored by the Synergy H1 microplate reader (BioTek, USA).

### 2.11. IRES Activity Assay

In accordance with established procedures, the construction of pRHF-CV-A9 and pRHF-CV-B3 (the vector plasmid was gifted by Dr. Zhen Luo, Jinan University, Guangdong), which includes CV-A9-5′-UTR or CV-B3-5′-UTR, positioned between the FLuc and RLuc genes, was carried out in the laboratory.

The pRHF-CV-A9 and pRHF-CV-B3 plasmids were transfected into HEK293T cells. After 4 h, serial concentrations of the compounds were added into each well. Cell lysates were collected and analyzed to determine FLuc and RLuc activity using a dual-luciferase reporter assay system (Yeasen Biotechnology (Shanghai) Co., Ltd., Shanghai, China) 48 h post-treatment following the manufacturer’s instructions.

### 2.12. In Vitro RNA Primer Extension Assay Using 3D^pol^

The 3D polymerase of CV-A9 required in the experiment was also expressed in *E. coli* BL21(DE3) using pET28a-3D recombinant plasmid constructed in our laboratory. A template RNA (5′-GGCGCACAAAGGUACCGUGAUACCAGUGUGCUGGCGCCCA-3′) was annealed to a primer (5′-FAM-CUGUGGGUUGUUCCCACCCA-3′). A primer RNA extension reaction was initiated and incubated with 1 mM NTPs and 1 μM RNA duplex to 3D^pol^ for 30 min in a buffer containing 2 U/µL RNasin, 4 mM MnCl_2_, 5 mM MgCl_2_, 5 mM DTT, 12.5 mM KCl, and 50 mM HEPES. Then it was quenched by adding quenching buffer (95% formamide, 20 mM EDTA, 0.01% bromophenol blue) and incubated at 95 °C for 10 min. The products were loaded onto 20% urea-denaturing polyacrylamide gel electrophoresis and separated at 200 V. Images were captured using a Tanon 5200 system (Shanghai Tanon Science & Technology Co., Ltd., Shanghai, China).

### 2.13. Molecular Docking

Docking was analyzed using AutoDock 4.2.6 and PyMOL 2.5.5. The CV-B3 3C^pro^ (PDB ID: 2ZTZ) and EV-A71 3C^pro^ (PDB ID: 4GHQ) structures were obtained from PDB (https://www.rcsb.org/) in pdb format. And the CV-A9 3C^pro^ structure model was based on the CV-B3 3C^pro^ structure (PDB ID: 2ZTZ), using the SWISS-MODEL server to generate it (https://swissmodel.expasy.org/, accessed on 11 November 2025). The structures of velutin (Compound CID: 5464381), isorhamnetin (Compound CID: 5281654), and (−)-epicatechin gallate (Compound CID: 107905) were obtained from PubChem (https://pubchem.ncbi.nlm.nih.gov/, accessed on 11 November 2025) in sdf format.

### 2.14. Cytokine Expression Analysis

To investigate the impact of drug treatment on the substantial upregulation of cytokines induced by viral infection, four distinct experimental groups were established: (i) RD cells treated with 50 μM compounds; (ii) RD cells infected with CV-A9 and treated with 50 μM compounds; (iii) RD cells infected with CV-A9; and (iv) RD cells without any treatment. The MOI value for CV-A9 was 0.001, and cell lysates were collected at 24 h.p.i. for RT-qPCR. The primer sequences for cytokines are listed in Table S3.

### 2.15. Antiviral Evaluation In Vivo

The animal experiments were approved by the Animal and Welfare Committee of Tianjin University (approval number: TJUE-2024-053). Four groups were established in this experiment. Seven-day-old BALB/c-WT mice were intraperitoneally injected with 50 μL of EV-A71 (6 × 10^5^ pfu/mL). HSBDF, velutin, and the positive control drug YM-201636 (a specific inhibitor of phosphoinositide kinase, FYVE finger containing (PIKFYVE) kinase, which was found to significantly suppress EV-A71 replication and virus-induced inflammation both in vitro and in vivo) [25] were diluted in PBS and administered intraperitoneally at a daily dose of 1.5 mg/g [26], 12.5 mg/kg, and 12.5 mg/kg per mouse, starting the day before infection in the treatment group. Meanwhile, the negative control group was treated with PBS. Animals were euthanized and dissected on the sixth day of infection. The quantification of viral load in tissues was analyzed by RT-qPCR. Pathological analysis of colon and heart tissues was performed using H&E staining.

### 2.16. Statistical Analysis

Statistical analysis was performed using GraphPad Prism 8. An unpaired *t*-test was used to evaluate the difference between the two groups. *p*-values ≤ 0.05 were regarded as significant for the analysis. * *p* < 0.05; ** *p* < 0.01; *** *p* < 0.001; **** *p* < 0.0001; ns: no significant difference.

## 3. Results

### 3.1. HSBDF Broadly Inhibits Enterovirus Infection

**Figure 1:**
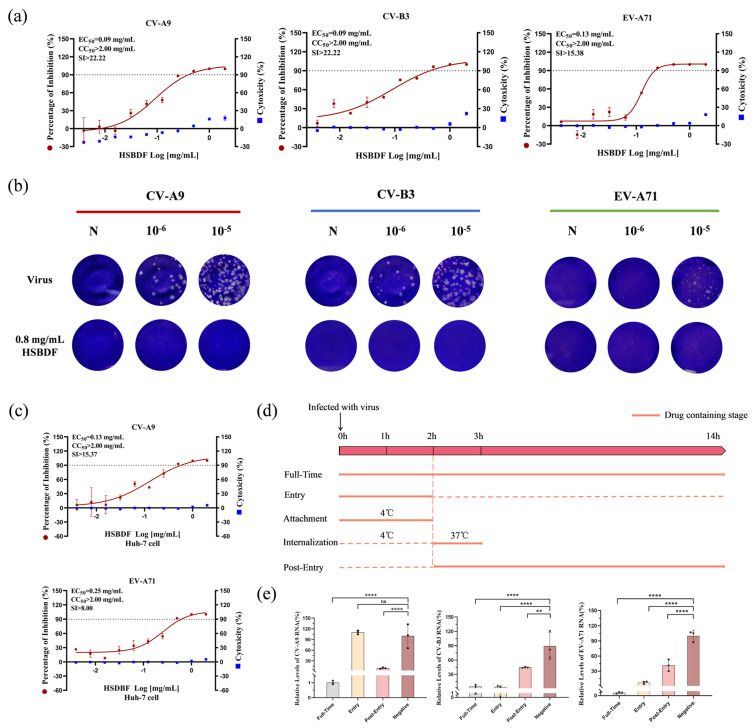
HSBDF effectively inhibited CV-A9, CV-B3, and EV-A71. (**a**) Dose-dependent antiviral activity of HSBDF against CV-A9 (**left**, RD cells), CV-B3 (**middle**, Vero E6 cells), and EV-A71 (**right**, RD cells) (left y-axis), with parallel cytotoxicity profiles (right y-axis). Antiviral activity was analyzed by quantifying viral nucleic acid load in cell lysates; cytotoxicity was assessed via the CellTiter-Blue cell viability assay. (**b**) Viral particle titers of CV-A9 (**left**, RD cells), CV-B3 (**middle**, Vero E6 cells), and EV-A71 (**right**, RD cells) in supernatants post-0.8 mg/mL HSBDF-treatment, quantified by plaque assay. (**c**) Dose-dependent antiviral activity of HSBDF against CV-A9 (**upper**) and EV-A71 (**lower**) in Huh-7 cells (left y-axis), with corresponding cytotoxicity (right y-axis). (**d**) Schematic of the time-of-addition assay design. Full-Time: cells were incubated with the drug-virus mixture throughout the infection period. Entry: cells were incubated with the drug-virus mixture for 2 h and washed 2 h after viral infection. Post-Entry: HSBDF was added 2 h after viral infection. Cells were collected for RT-qPCR detection at 12 h post-infection. (**e**) Intracellular viral RNA levels of CV-A9 (**left**), CV-B3 (**middle**), and EV-A71 (**right**) under HSBDF treatment at distinct lifecycle stages (time-of-addition assay). Representative curves of at least two independent experiments and presented as mean ± SD of three technical replicates. ns, no significant difference; ** *p* < 0.01; **** *p* < 0.0001.

We initially evaluated the antiviral activity of HSBDF against CV-A9 infection in RD cells. The results demonstrated potent viral suppression with 90% inhibition at 0.13 mg/mL (EC_50_ = 0.09 mg/mL) and negligible cytotoxicity (CC_50_ > 2.00 mg/mL, selectivity index [SI] > 22.22) (Figure 1a). Furthermore, the formulation exhibited broad-spectrum efficacy against multiple enteroviruses, including CV-B3, EV-A71, PV1, and Echo-11, with EC_50_ ranging from 0.09 mg/mL to 0.42 mg/mL (Figure 1a and Figure S1). Plaque assays were used to confirm its capacity to suppress progeny virion production of enterovirus at non-cytotoxic concentrations. HSBDF treatment reduced CV-A9 and CV-B3 titers by up to >5-log10 and that of EV-A71 by 4-log10 (Figure 1b). Subsequent validation in Huh7 cells revealed consistent antiviral potency across cell types (CV-A9: EC_50_ = 0.13 mg/mL; EV-A71: EC_50_ = 0.25 mg/mL) (Figure 1c), with efficacy maintained under varying MOI conditions (Figure S2). These findings collectively indicate that HSBDF dose-dependently suppresses enteroviral infections within its non-toxic concentration range through mechanisms independent of cellular specificity.

Additionally, time-of-addition assays revealed stage-specific antiviral mechanisms of HSBDF (Figure 1d). The formulation demonstrated pan-cycle inhibition against CV-B3 and EV-A71 infections, while selectively targeting post-entry stage in CV-A9 infection (Figure 1e). Consistent with these findings, viral attachment and internalization assays showed 0.8 mg/mL HSBDF significantly attenuated EV-A71 attachment (86.24% reduction) and cellular entry (76.17% inhibition) yet exhibited no interference with that of CV-A9 (Figure S3a,c). Subsequent viral replication kinetics analysis confirmed potent suppression of both viruses’ post-entry amplification (Figure S3b,d).

### 3.2. Transcriptome Analysis of HSBDF for the Treatment of CV-A9 Infection

**Figure 2:**
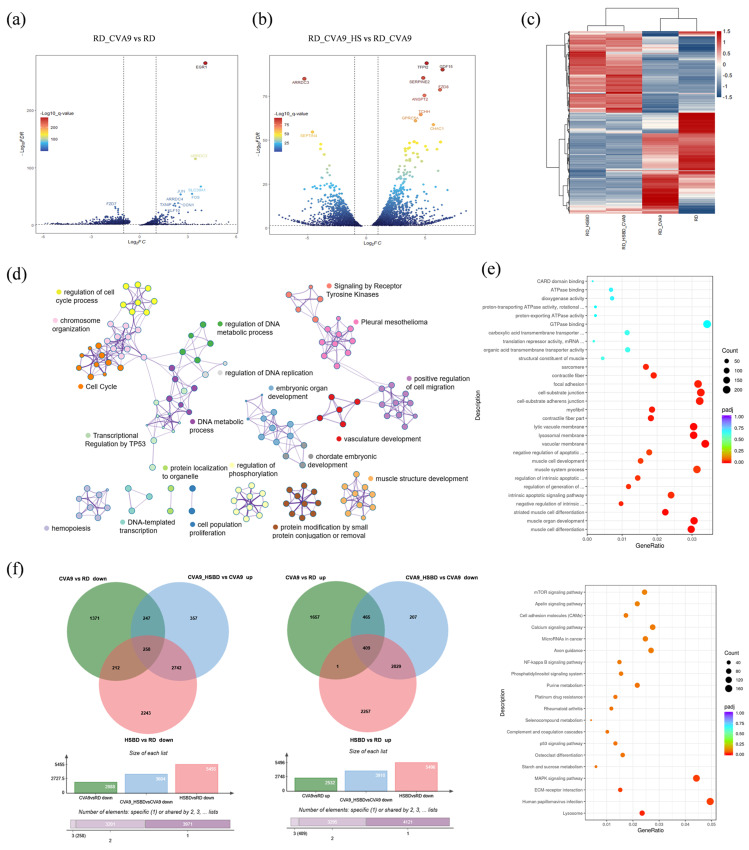
Transcriptome analysis of HSBDF against CV-A9 infection in RD cells. (**a**) Volcano plot of DEGs between CV-A9-infected RD cells and RD cells. (**b**) Volcano plot of DEGs between CV-A9-infected RD cells and CV-A9-infected RD cells with HSBDF treatment. The concentration of HSBDF was 1 mg/mL. (**c**) Heatmap of DEGs between the four groups. (**d**) Network of enriched terms colored by cluster ID, where nodes that share the same cluster ID are typically close to each other. (**e**) GO analysis (**above**) and KEGG pathway enrichment (**below**) of DEGs in CV-A9-infected cells after HSBDF treatment. (**f**) Venn diagram reflecting overlapping genes. Green indicates DEGs between CV-A9-infected RD cells and RD cells. Blue indicates DEGs between CV-A9-infected RD cells with HSBDF treatment and CV-A9-infected RD cells. Red indicates DEGs between RD cells with HSBDF treatment and RD cells.

Next, the analysis of the transcriptomic data pertaining to the formula treatment for CV-A9 level was conducted. Based on the data, we screened the entire dataset with the following standards: |Log2(Fold change)| ≥ 2, Q.value < 0.05. We plotted a differentially expressed gene volcano map (Figure 2a,b). The results indicated that some genes exhibited a very notable differential expression, being upregulated post-virus infection and subsequently downregulated following treatment administration. Some of these genes were all directly or indirectly involved in regulating multiple inflammatory signaling pathways [27,28,29]. This means that HSBDF may have a reverse regulatory effect on the inflammatory response after viral infection.

Furthermore, we overlapped the differentially expressed genes between the viral infection group and the HSBDF treatment group and found 2251 genes. This indicates that these viral infection-induced DEGs were further transcriptionally modulated by HSBDF treatment. We also performed a cluster analysis detailing the expression of differential genes in each comparator group (Figure 2c). Based on this result, pathway and process enrichment analysis was further performed. To further capture the relationships between the terms, a subset of enriched terms was selected and rendered as a network plot, where terms with a similarity > 0.3 are connected by edges. We selected the terms with the best *p*-values from each of the 20 clusters, and the network is visualized using Cytoscape (Figure 2d).

For group “RD_CV-A9_HSBDF vs. RD_CV-A9”, we analyzed the GO enrichment analysis from three perspectives: molecular function (MF), biological process (BP), and cellular components (CC). And KEGG pathway enrichment analysis was also carried out (Figure 2e). The analytical results showed that, with the addition of drugs to viral infection compared to control samples without drugs, most of the differential genes were significantly enriched in the signaling pathways of “mitogen-activated protein kinase (MAPK) signaling pathway” and “NF-kappa B signaling pathway”, which play important roles in inflammation, immunity, and stress responses [28,30]. And cluster analysis conducted on related gene expressions in each comparison group also confirmed the reversible regulatory effect of HSBDF (Figure S4).

Furthermore, Venn diagrams were used to identify the overlapping segments among genes downregulated by viral infection, genes upregulated by drug treatment alone, and genes upregulated in the drug-treated infection group (Figure 2f, left). This analysis aimed to delineate the subset of differentially expressed genes in the treatment group that were specifically attributable to the drug intervention. A total of 258 genes were identified within this overlapping set. Pathway enrichment analysis revealed that a proportion of these upregulated genes were implicated in the PI3K-AKT and TNF signaling pathways (Figures S5 and S6). Similarly, genes that were downregulated following viral infection but subsequently upregulated upon drug treatment (Figure 2f, right) were found to be associated with the MAPK signaling pathway (Figure S7), thereby corroborating our earlier analytical findings. To corroborate these observations, we quantified interferon mRNA levels in RD and 293T cells before and after pharmacological treatment of virus-infected or non-treated cultures. The data demonstrated that HSBDF attenuates the virus-induced upregulation of interferon expression in a dose-dependent manner (Figure S8).

### 3.3. Screening of Effective Antiviral Components from HSBDF

To elucidate the broad-spectrum anti-enteroviral constituents of HSBDF, we conducted high-throughput characterization of the chemical compounds in HSBDF through HPLC-MS/MS analysis. As shown in Figure S9a,b, the total ion current (TIC) diagram of HSBDF was classified into two categories based on the negative and positive ion modes of HPLC-MS/MS. The chemical components of HSBDF were identified using secondary mass spectrometry data with the Thermo mzCloud online database and Thermo mzValut local database. Overall, a total of 151 chemical compounds, including 41 flavonoids, 25 organooxygen compounds, 21 prenol lipids, 14 carboxylic acids and derivatives, 11 fatty acyls, 5 tannins, 4 benzene and substituted derivatives, 4 anthracenes, 3 phenols, 2 furopyrans, 2 hydroxy acids and derivatives, and 23 others were identified in HSBDF (Figure S9c).

**Figure 3:**
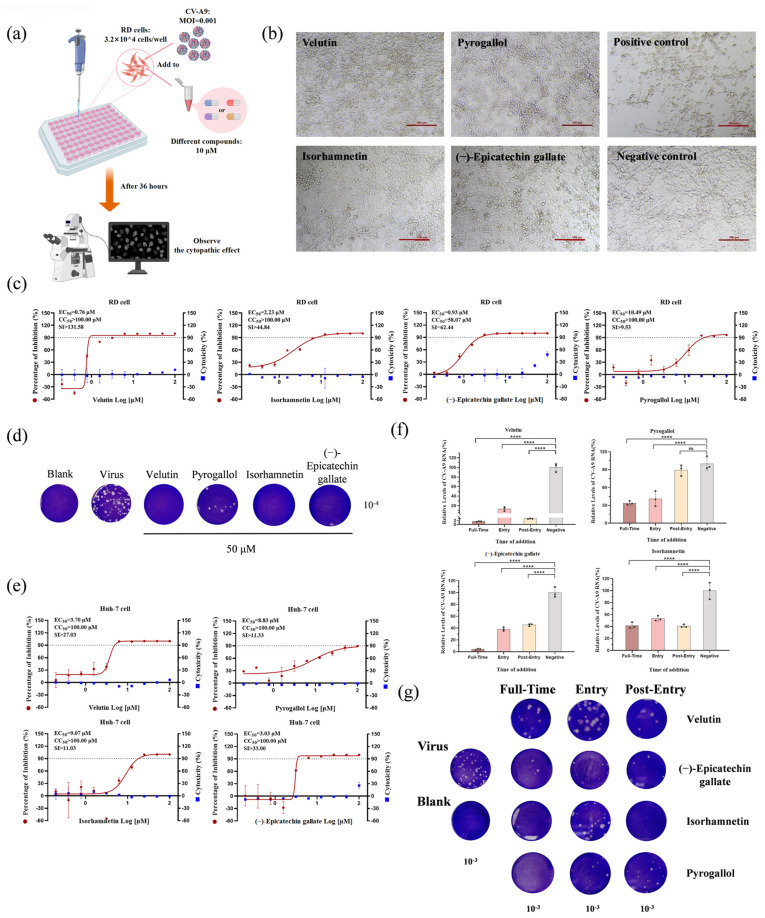
Evaluation of effective antiviral components of HSBDF at CV-A9 level. (**a**) Schematic diagram of screening for effective antiviral components. A total of 119 commercially available components obtained by mass spectrometry analysis of HSBDF were used for anti-CV-A9 activity evaluation. (**b**) Cytopathic effect (CPE) morphology of RD cells infected with CV-A9 (MOI = 0.001) and treated with compounds (10 μM) for 36 h. Positive control: CV-A9 infection without treatment; negative control: uninfected and untreated cells. (**c**,**e**) Dose–response curves of velutin, pyrogallol, isorhamnetin, and (−)-epicatechin gallate against CV-A9 in RD cells (**c**) and Huh 7 cells (**e**) (left y-axis: inhibition; right y-axis: cytotoxicity). (**d**) Viral titers in supernatants after 50 μM compound treatment were quantified by plaque assay. (**f**) Time-of-addition assays of CV-A9 (MOI = 0.001) for four compounds (25 μM) on RD cells. Cells were collected for RT-qPCR detection at 12 h post-infection. ns, no significant difference; **** *p* < 0.0001 (**g**) The plaque assay of supernatants from the time-of-addition assays of Figure (**f**); a 10^−3^ gradient is selected in the figure for comparison. Representative curves of at least two independent experiments presented as mean ± SD of three technical replicates.

Based on the principle of accessibility, we established an HSBDF monomeric compound library comprising 119 commercially available components. And then, a primary high-throughput screening for antiviral activity was performed by CV-A9-infected RD cell model. There, four compounds at 10 μM (velutin, pyrogallol, isorhamnetin, and (−)-epicatechin gallate) were identified as CV-A9 inhibitors by observing the CV-A9-induced CPEs (Figure 3a,b). It is noteworthy that velutin and pyrogallol have not been documented as agents with anti-enterovirus properties prior to this study. Subsequent dose–response characterization on RD cell lines revealed that velutin was the most promising candidate, exhibiting potent antiviral activity (EC_50_ = 0.76 μM) with minimal cytotoxicity (CC_50_ > 100 μM, SI > 131.58) (Figure 3c,d), with a relatively large therapeutic window and good druggability. The four compounds maintained significant anti-CV-A9 activity in Huh7 cells, SI remaining within clinically relevant therapeutic windows, albeit with marginally reduced potency compared to that in RD cells (Figure 3e).

**Figure 4:**
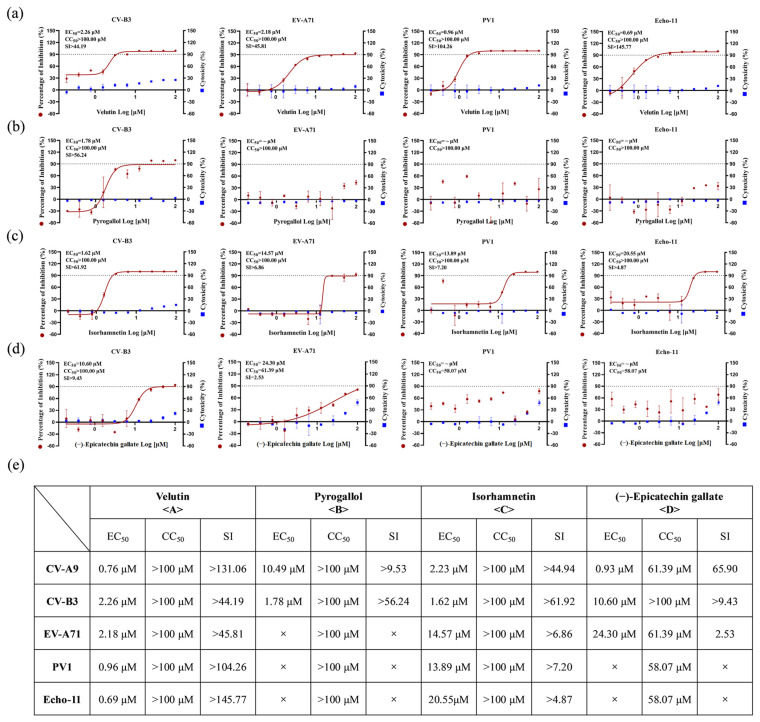
Inhibitory activities of bioactive compounds against other important enteroviruses. Dose–response curves of velutin (**a**), pyrogallol (**b**), isorhamnetin (**c**), and (−)-epicatechin gallate (**d**) against CV-B3 (MOI = 0.001), EV-A71 (MOI = 0.1), PV1 (MOI = 0.003), Echo-11 (MOI = 0.005). Left y-axis: inhibition; right y-axis: cytotoxicity. Representative curves of at least two independent experiments presented as mean ± SD of three technical replicates. (**e**) Summary of anti-enterovirus activity of velutin, pyrogallol, isorhamnetin, and (−)-epicatechin gallate, × indicated no antiviral effect at concentrations below 100 μM.

The broad-spectrum anti-enteroviral activity of these four compounds was systematically validated. Velutin demonstrated superior efficacy against multiple enterovirus species (CV-B3, EV-A71, PV1, and Echo-11), exhibiting exceptional selectivity indices (SI: >44.19 to >145.77) (Figure 4a,e). Isorhamnetin exhibited comparable antiviral breadth, albeit with lower antiviral potency than velutin (SI: >4.87 to >61.92) (Figure 4c). In contrast, pyrogallol showed selective inhibition against CV-A9 and CV-B3 infections (Figure 4b), and (−)-epicatechin gallate exhibited limited spectrum activity restricted to CV-A9, CV-B3, and EV-A71 infections, with better activity against coxsackievirus than against EV-A71 (Figure 4d).

### 3.4. Bioactive Compounds Exhibit Broad-Spectrum Pan-Cycle Inhibition of Enteroviral Infection

To elucidate the antiviral mechanisms of four candidate compounds, time-of-addition assays were systematically conducted against CV-A9 infection. Results revealed distinct phase-specific activity: pyrogallol exclusively targeted viral attachment, whereas velutin, isorhamnetin, and (−)-epicatechin gallate exhibited pan-stage suppression spanning both entry and post-entry phases (Figure 3f). This mechanistic divergence potentially accounts for pyrogallol’s comparatively higher EC_50_ values versus other candidates. Plaque quantification of supernatants substantiated these findings, demonstrating a significant reduction in infectious particles after treatment with velutin, isorhamnetin, and (−)-epicatechin gallate at both the entry and post-entry stages (Figure 3g). Furthermore, although the addition of pyrogallol after the virus enters the cells cannot effectively inhibit the proliferation of viral RNA in the cells, it still has a certain inhibitory activity on the release of progeny viral particles (Figure 3g).

Complementary attachment/internalization assays further delineated velutin’s complete blockade of viral entry (attachment: 40.54% inhibition; internalization: 38.24% at 25 μM), contrasting with pyrogallol’s restriction to attachment phase inhibition (32.43%) without affecting cellular internalization (Figure S3e,f). Additionally, viral load kinetic analyses at post-entry confirmed velutin significantly suppressed intra- and extracellular viral proliferation throughout infection cycles, though it was less potent than CHX (positive control drug) (Figure S3g).

Next, we preliminarily evaluated the interactions of four active monomeric compounds using a combination therapy strategy. Drug combination effects were visualized and synergistic interactions were quantified via the Zero Interaction Potency (ZIP) model on the SynergyFinder platform (https://synergyfinder.fimm.fi, accessed on 11 November 2025) [31,32]. Results revealed additive inhibitory effects (ZIP scores: 1.684, 3.126, and 1.443, respectively) between velutin and pyrogallol, isorhamnetin, or (−)-epicatechin gallate (Figure S10), suggesting that these compounds may exert biological effects through shared molecular targets.

### 3.5. Bioactive Compounds Targets the Conserved 3C Protease to Suppress Enteroviral Replication

In the life cycle of enteroviruses, the 2A protease (2A^pro^), 3C protease (3C^pro^), 3D protease (3D^pol^), and internal ribosome entry site (IRES) are recognized as critical targets for antiviral drug development [1]. Specifically, 2A^pro^ and 3C^pro^ cleave the viral polyprotein precursor (P1–P3) into functional proteins while disrupting host gene expression to facilitate viral replication [33,34,35]. The 3D^pol^ forms the RNA-dependent RNA polymerase (RdRp) essential for viral RNA synthesis [36], whereas the IRES drives cap-independent translation initiation by recruiting eukaryotic translation initiation factors [37].

**Figure 5:**
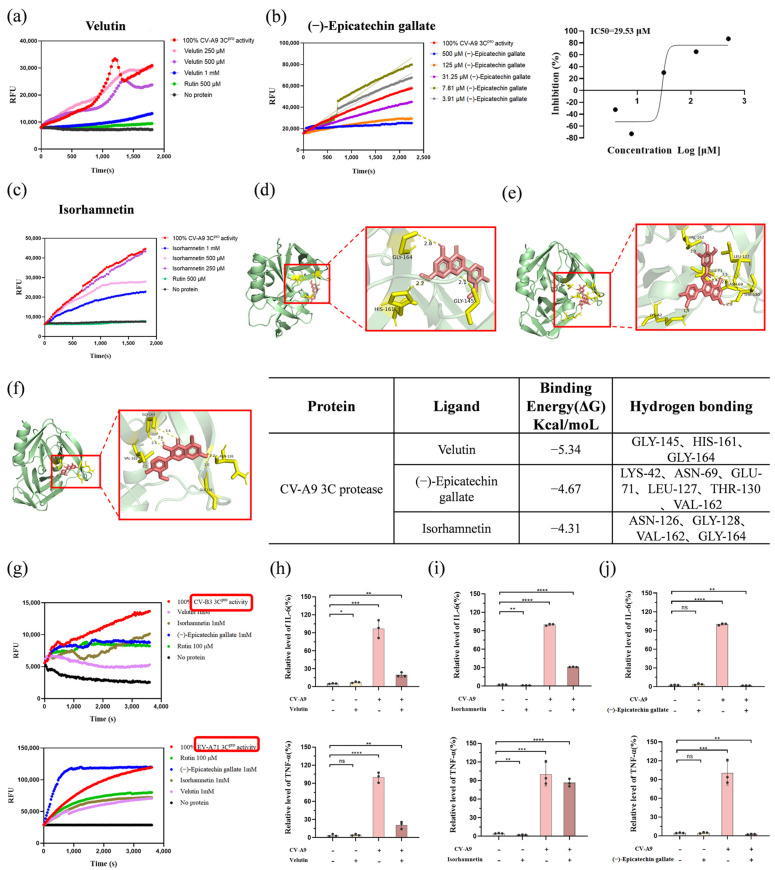
Velutin, isorhamnetin, and (−)-epicatechin gallate inhibited enterovirus 3C^pro^ enzyme activities and alleviated high expression of inflammatory factors. (**a**–**c**) Effect of different concentrations of velutin (**a**), (−)-epicatechin gallate (**b**), and isorhamnetin (**c**) on CV-A9 3C^pro^ cleavage activity in vitro, with rutin as the positive control. (**d**–**f**) Molecular docking of CV-A9 3C^pro^ (green surface) with velutin (**d**), (−)-epicatechin gallate (**e**), and isorhamnetin (**f**). Yellow dashed lines denote hydrogen bonds and attached residues; interacting residues and binding energies (ΔG) are tabulated. (**g**) Inhibitory effects of velutin, isorhamnetin, and (−)-epicatechin gallate on in vitro cleavage activity of CV-B3 and EV-A71 3C^pro^ (rutin as positive control). (**h**–**j**) mRNA expression levels of IL-6 (**top**) and TNF-α (**bottom**) in CV-A9-infected cells (MOI = 0.001) treated with 50 μM velutin (**h**), isorhamnetin (**i**), and (−)-epicatechin gallate (**j**) for 24 h. Data mean ± SD of three technical replicates. ns, no significant difference; * *p* < 0.05; ** *p* < 0.01; *** *p* < 0.001; **** *p* < 0.0001.

Given the potent inhibitory effects of velutin, isorhamnetin, and (−)-epicatechin gallate on CV-A9 replication across its entire life cycle, we systematically evaluated their capacity to modulate the enzymatic functions of 2A^pro^, 3C^pro^, 3D^pol^, and IRES elements in vitro. The enzymatic functions of 2A^pro^, 3C^pro^ were conducted by a FRET-based protease assays with the known 3C^pro^ inhibitor rutin serving as a positive control and the compound and protease were pre-incubated at 25 °C for 30 min prior to the addition of the substrate. The enzymatic functions of 3D^pol^ conducted in vitro polymerase activity assays to assess the efficacy of three compounds, utilizing ethylisopropylamiloride (EIPA) as a positive control. 293T cells were treated with different concentrations of compounds for 36 h. Cell lysates were then collected for RLuc and Fluc activity analysis to evaluate IRES activity. Biochemical analyses revealed that velutin, isorhamnetin, and (−)-epicatechin gallate exhibited no inhibitory activity against 2A^pro^, 3D^pol^, and IRES elements (Figure S11a–c). However, all three compounds demonstrated significant inhibition of CV-A9 3C^pro^ activity, with (−)-epicatechin gallate displaying the most potent inhibitory effect (IC_50_ = 29.53 μM; Figure 5a–c). Building upon their broad-spectrum antiviral efficacy against CV-A9, CV-B3, and EV-A71 strains, we subsequently investigated these compounds’ ability to attenuate 3C^pro^ activity of CV-B3 and EV-A71. The results showed that 1 mM of (−)-epicatechin gallate and isorhamnetin inhibited about 50% of the protease activity; meanwhile, velutin made it almost impossible for the protease to cleave the substrate peptide at the same concentration (Figure 5g, top). This result does not seem to be surprising, as the amino acid homology of 3C^pro^ between CV-B3 and CV-A9 strains can be as high as 99%. Additionally, isorhamnetin and velutin demonstrated favorable inhibitory effects on 3C^pro^ of EV-A71, whereas (−)-epicatechin gallate exhibited no activity in a corresponding manner (Figure 5g, bottom).

Molecular docking analysis revealed the binding mode of bioactive compounds to the 3C^pro^. Velutin exhibited the strongest binding affinity (ΔG = −5.34 kcal/mol), forming hydrogen bonds with GLY-145, HIS-161, and GLY-164 within the catalytic pocket (Figure 5d). (−)-Epicatechin gallate (ΔG = −4.67 kcal/mol) engaged a broader network of polar interactions, including the residues LYS-42, ASN-69, GLU-71, LEU-127, THR-130, and VAL-162 (Figure 5e). Isorhamnetin (ΔG = −4.31 kcal/mol) primarily bound via ASN-126, GLY-128, VAL-162, and GLY-164 (Figure 5f), suggesting preferential stabilization through β-sheet adjacent regions. These interactions collectively indicate that hydrogen bonding with conserved residues (e.g., GLY-164) and catalytic triad-proximal motifs underpins the differential inhibitory potency of these flavonoids against CV-A9 3C^pro^. Notably, velutin, isorhamnetin, and (−)-epicatechin gallate exhibited hydrogen bonding with the conserved GLY-166 residue in CV-B3 3C^pro^ (Figure S12a–d). Moreover, velutin and isorhamnetin occupied the GLN-42-associated subsite within the EV-A71 3C^pro^ catalytic domain, destabilizing a resistance-related spatial motif (Figure S12a–d). These findings demonstrate that these flavonoids suppress proteolytic activity via structural disruption of the 3C^pro^ catalytic architecture across enterovirus serotypes.

### 3.6. Bioactive Compounds Demonstrate Anti-Inflammatory Efficacy

Enteroviral infection can induce cytokine storm in a variety of tissues, which is characterized by marked upregulation of key inflammatory mediators, including cytokines (e.g., IL-6, TNF-α), chemokines (e.g., MCP-1), and COX-2, etc. [38]. Notably, IL-6 and TNF-α, serving as core pro-inflammatory cytokines that coordinate the activation of diverse immune cells [39], exhibit a significant positive correlation between their aberrantly elevated expression levels and the severity of the disease. Therefore, therapeutic strategies against enteroviruses should incorporate targeted interventions for cytokine storms. In this study, systematic evaluation using a CV-A9 infection model demonstrated significant upregulation of IL-6, TNF-α, MCP-1, and COX-2 (Figure 5h−j and Figure S13).

Pharmacological analyses further revealed that both velutin and (−)-epicatechin gallate exhibited inhibitory effects on CV-A9 infection-induced aberrant expression of inflammatory mediators. Specifically, 50 μM velutin significantly suppressed the upregulation of IL-6, TNF-α, and COX-2 (inhibition rates: 70–80%) yet showed limited efficacy against MCP-1 (20% inhibition) (Figure 5h and Figure S13). In parallel, 50 μM (−)-epicatechin gallate achieved 80–90% inhibition of IL-6, TNF-α, MCP-1, and COX-2 (Figure 5j and Figure S13). In contrast, isorhamnetin selectively attenuated IL-6 expression by about 60% at 50 μM without significant effects on other mediators (Figure 5i and Figure S13).

Our experimental data presented in this study demonstrate that treatment with the active compounds effectively suppressed the significant and harmful increase in cytokines/chemokines caused by CV-A9 infection, thereby potentially mitigating virus-induced cytokine storms.

### 3.7. Active Compounds Inhibit Viral Infection in Suckling Mouse Model

**Figure 6:**
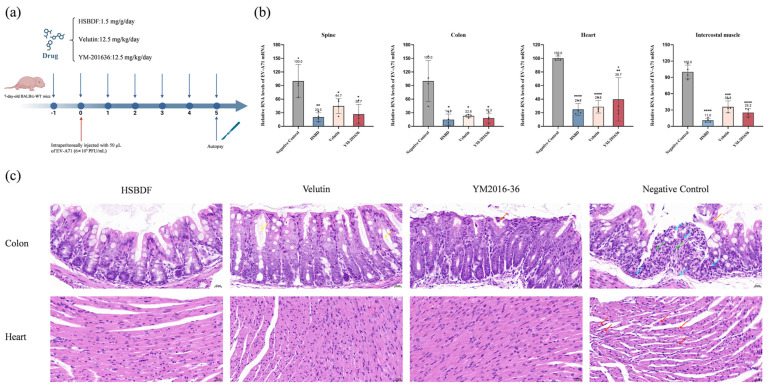
HSBDF and velutin effectively inhibited EV-A71 in vivo in the BALB/c mouse model. (**a**) Schematic diagram of the animal experiment. Seven-day-old BALB/c WT mice were intraperitoneally injected with 50 μL EV-A71 per mouse and separately treated intraperitoneally with PBS or compounds. At 5 d.p.i., the viral loads in various tissues of mice were quantified by qRT-PCR. (**b**) Viral load in spine, colon, heart, or intercostal muscle for each group. ns, no significant difference; * *p* < 0.05; ** *p* < 0.01; *** *p* < 0.001; **** *p* < 0.0001. (**c**) The intestines/heart of mice were stained with hematoxylin and eosin (H&E). The orange arrow indicates epithelial cell detachment; the green arrow indicates intestinal gland necrosis/disappearance, replaced by connective tissue hyperplasia; the blue arrow indicates scattered infiltration of lymphocytes and granulocytes; the yellow arrow indicates the intestinal gland dilatation; and the red arrow indicates vacuolar degeneration of cardiomyocytes.

To assess the efficacy of the compounds in inhibiting enterovirus infection in vivo, BALB/c mice were utilized in the study [25]. Seven-day-old mice in the experiment were challenged with EV-A71 and treated with the drug at the specified daily dose from the day prior to viral challenge, with YM-201636 treatment as a positive control. All mice were executed and dissected on day 5 post-infection (Figure 6a). Viral loads in the spine, colon, heart, and intercostal muscle were quantified by RT-qPCR. As shown in Figure 6b, treatment with the compounds resulted in a significant reduction in viral copy numbers of EV-A71.

Further histopathological analysis revealed that EV-A71 infection induced substantial damage in the intestine and heart. As shown in Figure 6c, intestinal section exhibited significant pathological changes, including shedding of epithelial cells (orange arrow) and infiltration of inflammatory cells (lymphocytes and granulocytes) in the submucosa (blue arrow). Furthermore, viral infection induced extensive necrosis and disappearance of a large number of intestinal glands, with their positions replaced by connective tissue hyperplasia (green arrow). Meanwhile, partial shedding of intestinal epithelial cells was observed in the YM-201636 group (orange arrow), and there was a mild reduction in the number of cup-shaped cells, with occasional intestinal gland dilatation (yellow arrow). However, no epithelial cell detachment was observed in the other drug-administration groups. Treatment with velutin and HSBDF resulted in no hydropic degeneration of the epithelial cells, and treatment with velutin caused mild dilatation of a small number of intestinal glands. In addition, EV-A71 infection caused the myocardium to show variation in cardiomyocyte size and loose and irregular arrangement, as well as vacuolar degeneration in a small number of cardiomyocytes (red arrow), which improved with drug treatment. In conclusion, our findings indicated that interventional therapy with HSBDF and velutin reduced viral loads in the spine, colon, heart, and intercostal muscle, as well as mitigated virus-induced histopathological damage in the intestine.

## 4. Discussion

This study demonstrates that the commercial HSBDF granule, a standardized traditional Chinese medicine formulation, exhibits broad-spectrum anti-enteroviral activity. Notably, HSBDF alleviates virus-induced inflammatory responses by suppressing NF-κB and MAPK signaling pathways, highlighting its clinical potential for managing enterovirus-associated pathologies. To address the longstanding challenge of undefined pharmacodynamic material basis in TCM modernization, we systematically characterized 152 compounds in HSBDF through HPLC-MS/MS analysis. Among 119 commercially available candidates, four bioactive constituents (velutin, pyrogallol, isorhamnetin, and (−)-epicatechin gallate) were identified as effective enterovirus inhibitors. Of significance, this study provides the first evidence of the anti-enteroviral properties of velutin and pyrogallol. Mechanistically, the three flavonoid-based compounds (velutin, isorhamnetin, and (−)-epicatechin gallate) exerted inhibitory effects across the entire viral life cycle, whereas the pyrogallol preferentially targeted viral entry. These findings underscore the superior antiviral potential of flavonoids against enteroviruses, providing a scientific foundation for developing structure-guided drug development.

Subsequent mechanistic studies revealed that none of the four active constituents significantly affected the activities of enteroviral 2A^pro^, 3D^pol^, or IRES elements. Notably, velutin, isorhamnetin, and (−)-epicatechin gallate exerted broad-spectrum anti-enteroviral effects by targeting the 3C^pro^. Consistently, multiple flavonoids—including luteoloside [40], quercetin [41], fisetin, and rutin [42]—have been reported to inhibit 3C^pro^ activity across enteroviral species. Although the precise molecular interactions between these compounds and enteroviral 3C^pro^ remain underexplored, the conserved flavonoid scaffold may engage conserved structural motifs critical for 3C^pro^ inhibition. Functionally, enteroviral 3C^pro^ acts as a pleiotropic enzyme, mediating viral polyprotein processing for replication and subverting host immunity by cleaving immune regulators (e.g., NF-κB, MAPK) to exacerbate pathogenesis [1,43,44]. Phylogenetic analysis further highlights the absence of mammalian homologs for 3C^pro^ [45], reinforcing its therapeutic specificity.

Consistent with the anti-inflammatory effects of HSBDF, three bioactive compounds (velutin, isorhamnetin, and (−)-epicatechin gallate) significantly attenuated virus-induced upregulation of inflammatory mediators. This observation aligns with established anti-inflammatory mechanisms of flavonoids, which suppress NF-κB nuclear translocation and inhibit p38/JNK MAPK phosphorylation, thereby downregulating pro-inflammatory cytokines (e.g., TNF-α, IL-6) [46,47]. Supporting this paradigm, anti-SARS-CoV-2 profiling of HSBDF [17] identified six viral inhibitors (four flavonoids) and three anti-inflammatory agents (all flavonoid derivatives), collectively underscoring the dual therapeutic role of flavonoids in targeting viral replication and inflammation-driven pathogenesis.

A notable limitation of this study lies in BALB/c mice, which possess robust innate and adaptive immunity, simply observing a reduction in viral load is insufficient to claim therapeutic utility. Therefore, the primary therapeutic value of HSBDF lies in its ability to attenuate excessive immunopathology—the harmful overactivation of the immune system—while still allowing for effective viral clearance. This immunomodulatory effect, reducing collateral tissue damage without compromising antiviral defense, represents a crucial complementary mechanism to the host’s intrinsic immunity and forms the basis for its potential as an adjunctive therapy.

Overall, our findings provide novel insights into TCM-based antiviral strategies against enteroviruses. Specifically, the rational design of broad-spectrum 3C^pro^ inhibitors leveraging flavonoid scaffolds, coupled with optimizing the botanical composition of HSBDF to enrich flavonoid-rich plant sources, emerges as a pivotal direction for advancing both phytopharmaceutical development and structure-based drug discovery.

## 5. Conclusions

In conclusion, this study systematically identifies the pharmacodynamic material basis of HSBDF granules, revealing its broad-spectrum anti-enteroviral activity and anti-inflammatory properties. We characterized 152 compounds and identified 4 key bioactive constituents, with velutin and pyrogallol reported for the first time as enterovirus inhibitors. Mechanistically, the flavonoid-based compounds (velutin, isorhamnetin, and (−)-epicatechin gallate) exert their broad-spectrum antiviral effects by targeting the highly conserved viral 3C^pro^, while simultaneously alleviating virus-induced inflammatory responses via suppression of the NF-κB and MAPK pathways. These findings not only elucidate the dual therapeutic role of flavonoids in combating viral replication and inflammation-driven pathogenesis but also underscore the 3C^pro^ as a promising and specific drug target.

Collectively, our work provides a scientific foundation for the modernization of HSBDF and offers strategic directions for future antiviral drug development. The rational design of broad-spectrum 3C^pro^ inhibitors based on the flavonoid scaffold, coupled with the optimization of the HSBDF formulation to enrich flavonoid content, represents a pivotal path forward for advancing both phytopharmaceutical development and structure-based drug discovery against enteroviruses.

## Data Availability

The original contributions presented in this study are included in the article/Supplementary Materials. Further inquiries can be directed to the corresponding authors.

## Supplementary Materials

The following supporting information can be downloaded at https://www.mdpi.com/article/10.3390/biology14111615/s1, Figure S1. The EC50 and CC50 evaluations of HSBDF anti PV1 andEcho-11. Figure S2. HSBDF inhibited CV-A9 in different cell lines at different MOIs. Figure S3. Specific phases of antiviral action of HSBDF, pyrogallol, and velutin. Figure S4. HSBDF modulated MAPK and NF-κB signaling pathways by reversing infection-driven gene dysregulation. Figure S5. HSBDF upregulated PI3K-AKT signaling pathways by reversing infection-driven gene dysregulation. Figure S6. HSBDF upregulated TNF signaling pathways by reversing infection-driven gene dysregulation. Figure S7. HSBDF downregulated MAPK signaling pathways by reversing infection-driven gene dysregulation. Figure S8. Effect of HSBDF on the expression of IFN-α, IFN-β and IFN-λ induced by CV-A9 infection on RD and 293T cells. Figure S9. Chemical identification of HSBDF using HPLC-MS/MS. Figure S10. Possibility of the combination of some compounds. Figure S11. Inhibitory activities of compounds on CV-A9 3D^pol^, IRES, and 2A^pro^. Figure S12. Docking analysis of compounds with CV-B3 and EV-A71 3C^pro^ models. Figure S13. Effect of bioactive compounds on the expression of MCP-1 and COX-2 induced by CV-A9 infection. Table S1. Full spectrum identification results of Huashi Baidu formula (positive ion model). Table S2. Full spectrum identification results of Huashi Baidu formula (negative ion model). Table S3. The relevant primer sequences mentioned in the article. Table S4. Data of the transcriptional difference between virus-treated RD cells and RD cells. Table S5. Data of the transcriptional difference between HSBD-treated RD cells and virus-treated RD cells.
