# Supplementary material for: HSBDF-Derived Bioactive Components Broadly Inhibit Enteroviruses by Targeting 3C Protease and Attenuating Inflammatory Responses
Source: Biology (Basel). 2025 Nov 18;14(11):1615. doi: 10.3390/biology14111615 (PMC12650729; doi:10.3390/biology14111615)
Supplement: Supplementary file 1 [file biology-14-01615-s001.zip › biology-3937341-supplementary.pdf]

HSBDF-Derived Bioactive Components Broadly Inhibit Enteroviruses by Targeting 3C Protease and Attenuating Inflammatory Responses

Ruolan Hu [1,†], Lin Guan [2,†], Siyue Li [2], Chunlin Liu [1], Gang Huang [2], Fuxing Lou [2], Hongzheng Jiang [2], Shuqi Wang [2], Zehan Pang [2], Yaxin Wang [1], Zhenlu Li [1], Han Zhang [3], Yigang Tong [2], Huahao Fan [1,2,3,4,5,*] and Bixia Hong [6,*]

[1] School of Life Sciences, Tianjin University, Tianjin 300072, China
[2] College of Life Science and Technology, Beijing University of Chemical Technology, Beijing 100029, China
[3] State Key Laboratory of Component-Based Chinese Medicine, Tianjin University of Traditional Chinese Medicine, Tianjin 301617, China
[4] State Key Laboratory of Synthetic Biology, Tianjin University, Tianjin 300072, China
[5] State Key Laboratory of Pathogen and Biosecurity, Beijing Institute of Microbiology and Epidemiology, Beijing 100071, China
[6] State Key Laboratory of Respiratory Disease, School of Basic Medical Science, Guangzhou Medical University, Guangzhou 511436, China
* Correspondence: fanhuahao@tju.edu.cn (H.F.); hongbixia@gzhmu.edu.cn (B.H.)
† These authors contributed equally to this work.

Figure S1

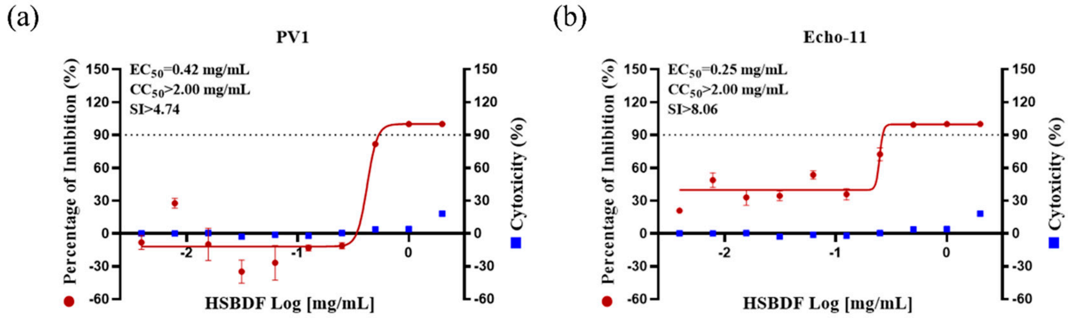

Figure S1. The EC_{50} and CC_{50} evaluations of HSBDF anti PV1 andEcho-11.

(a-b) Dose-dependent antiviral activity (left y-axis) of HSBDF against PV1 (MOI=0.003) **(a)** and Echo-11 (MOI=0.005) **(b)** on RD cells, with parallel cytotoxicity profiles (right y-axis). Antiviral activity was analyzed by quantifying viral nucleic acid load in cell lysates; cytotoxicity was assessed via the CellTiter-Blue cell viability assay. Representative curves of at least two independent experiments and presented as mean ± SD of three technical replicates.

Figure S2

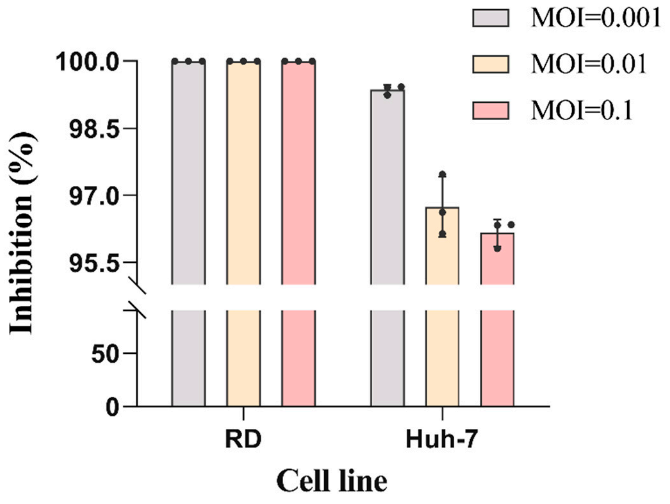

Figure S2. HSBDF inhibited CV-A9 in different cell lines at different MOIs.

RD cells or Huh-7 cells were infected with CV-A9 (MOI=0.001, 0.01, 0.1) and HSBDF (0.8 mg/mL). Cells were collected for RT-qPCR at 36 h.p.i, 30 h.p.i, and 24 h.p.i, respectively. Inhibition rates were calculated based on the CV-A9 load ratio of the treatment group/control. Representative curves of at least two independent experiments and presented as mean ± SD of three technical replicates.

Figure S3

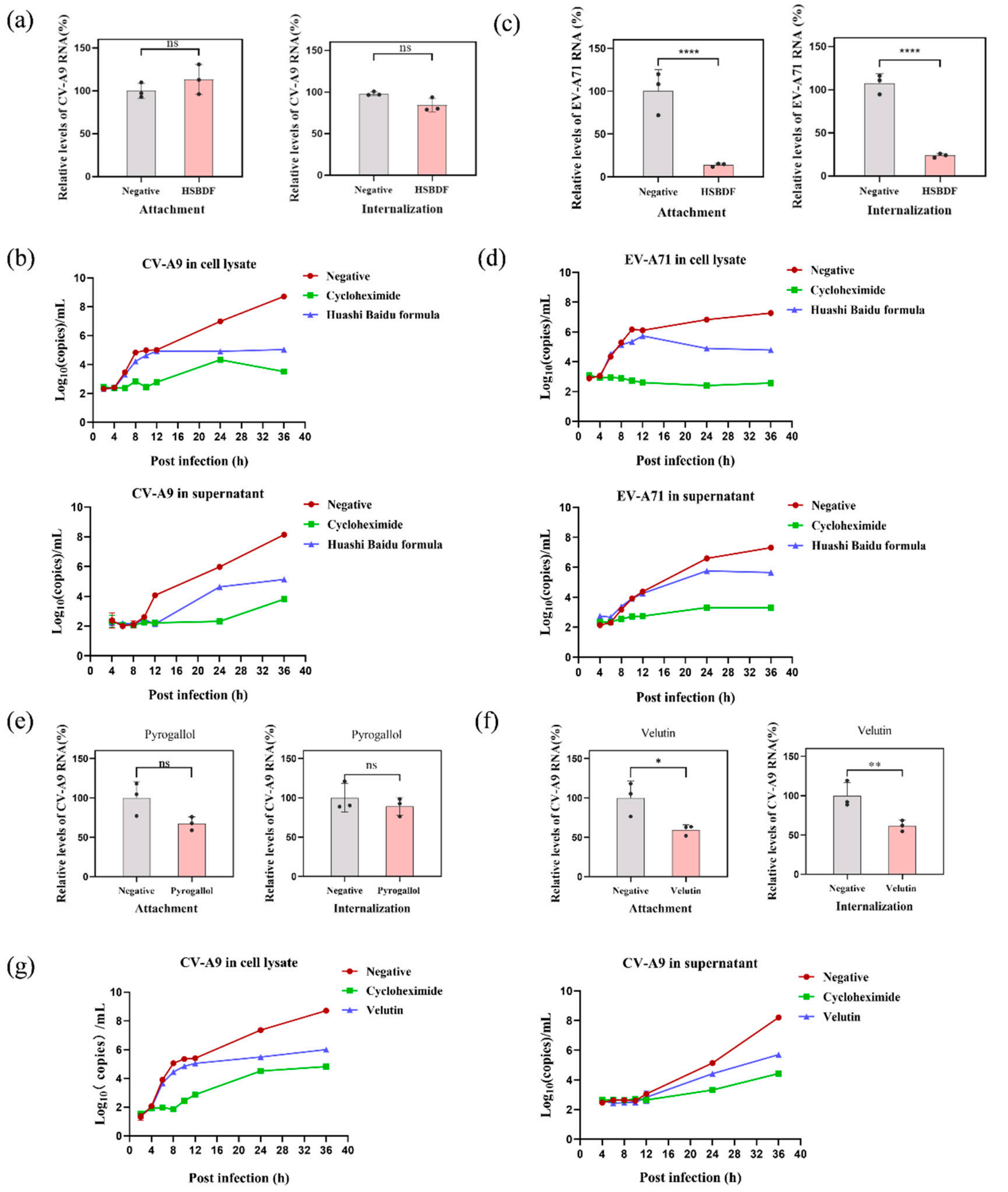

Figure S3. Specific phases of antiviral action of HSBDF, pyrogallol, and velutin. **(a, c)** Attachment and internalization assay of HSBDF (0.8 mg/mL) against CV-A9 infection (a, MOI=5) and EV-A71 infection (c, MOI=5). **(b, d)** Time-course suppression of viral replication by HSBDF. Viral RNA loads in cells (upper) and supernatants (lower) of CV-A9- (b, MOI=0.001) or EV-A71-infected (d, MOI=0.1) RD cells treated with 0.8 mg/mL HSBDF (added at 2 h post-infection). Cycloheximide (20 μM) served as the positive drug. **(e, f)** Pyrogallol (e) and velutin (f) at a concentration of 25 μM were assessed for blocking CV-A9 (MOI=5) attachment and internalization in RD cells. **(g)** Viral RNA loads of different time in cells (left) and supernatants (right)

of CV-A9-infected (MOI=0.001) RD cells treated with 25 μM velutin (added at 2 h post-infection), with cycloheximide (20 μM) as the positive drug. Representative curves of at least two independent experiments and presented as mean ± SD of three technical replicates. ns, no significant difference; ns, no significant difference; * $p < 0.05$; ** $p < 0.01$; *** $p < 0.001$; **** $p < 0.0001$.

Figure S4

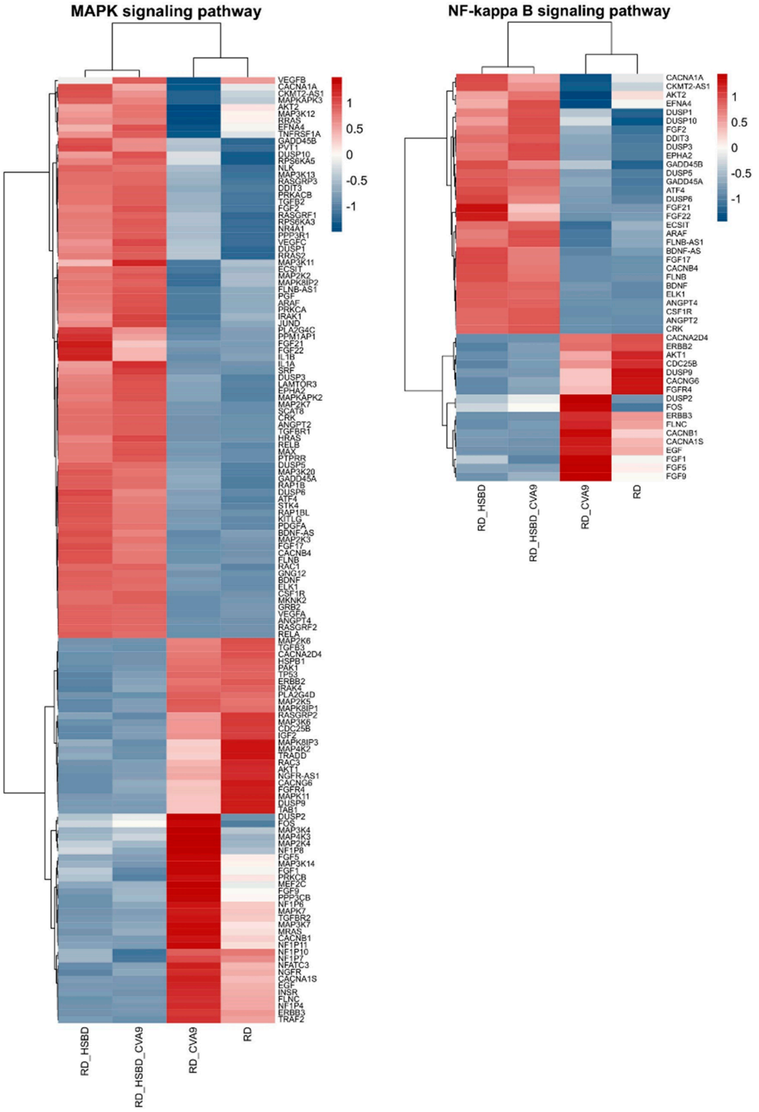

Figure S4. HSBDF modulated MAPK and NF-κB signaling pathways by reversing infection-driven gene dysregulation.

Heatmap of differentially expressed genes (DEGs) associated with MAPK and NF-κB signaling across four experimental groups (RD cells, CV-A9-infected, HSBDF-treated, and CV-A9-infected + HSBDF-treated).

Figure S5

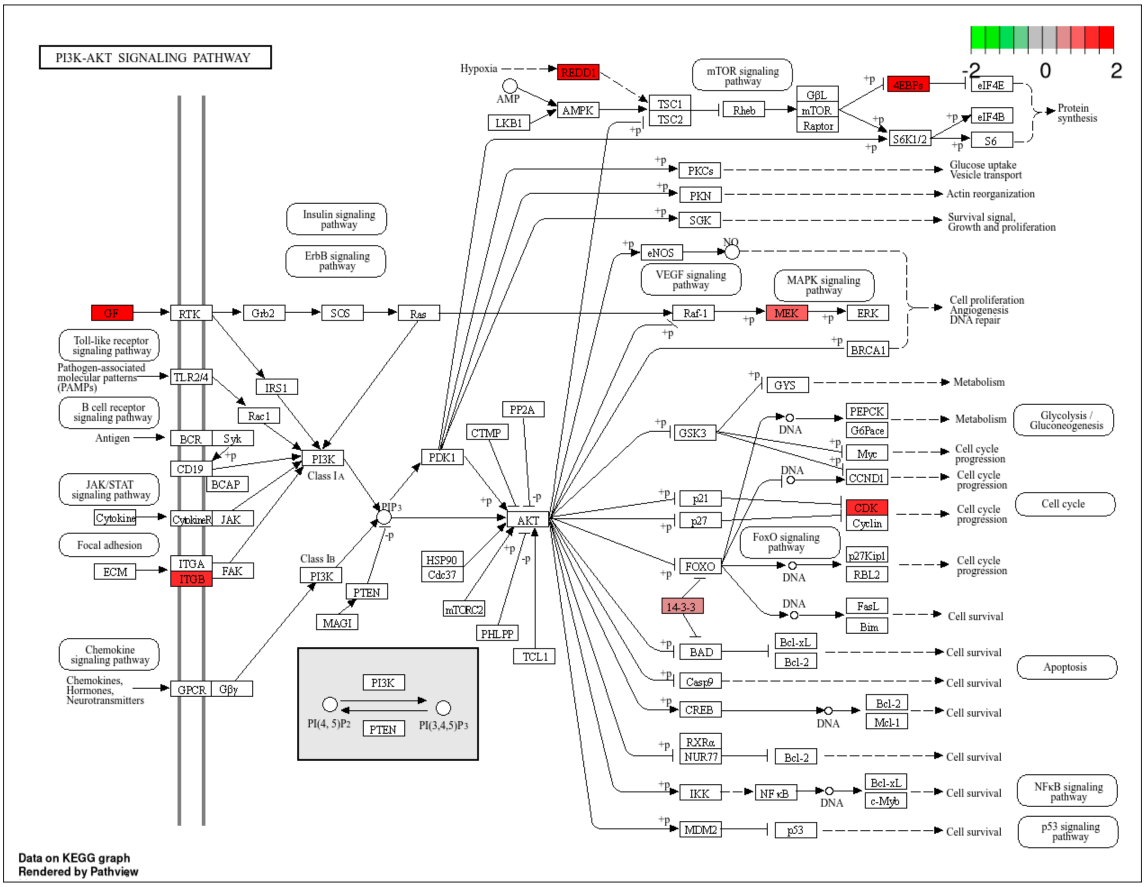

Figure S5. HSBDF upregulated PI3K-AKT signaling pathways by reversing infection-driven gene dysregulation.

The signaling pathway diagram of the overlapping genes related to the PI3K-AKT signaling pathway in the Venn diagram.

Figure S6

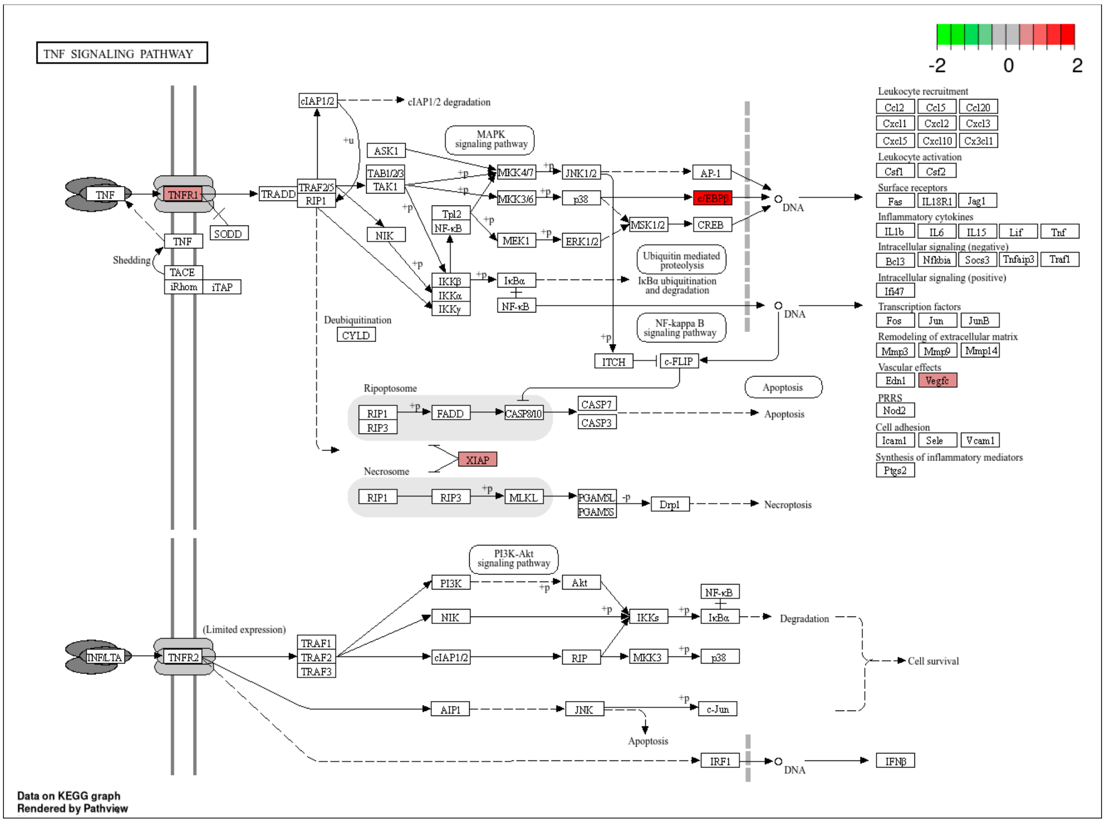

Figure S6. HSBDF upregulated TNF signaling pathways by reversing infection-driven gene dysregulation.

The signaling pathway diagram of the overlapping genes related to the TNF signaling pathway in the Venn diagram.

Figure S7

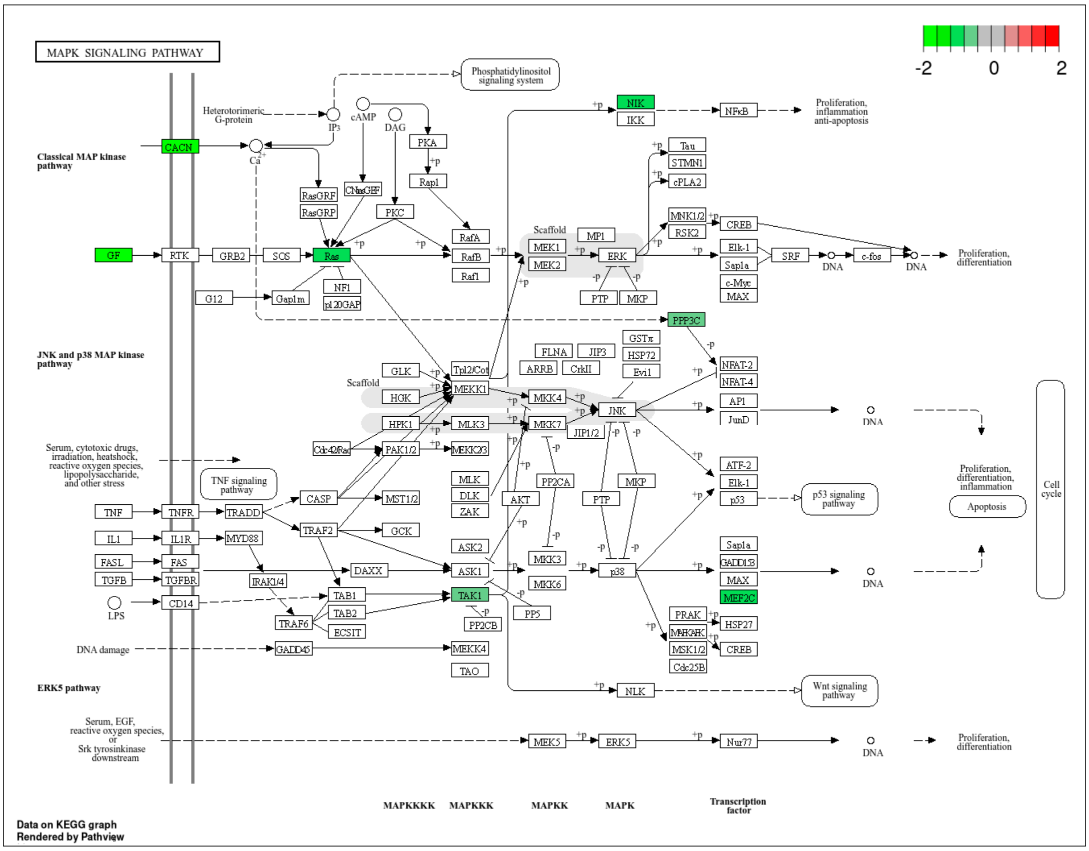

Figure S7. HSBDF downregulated MAPK signaling pathways by reversing infection-driven gene dysregulation.

The signaling pathway diagram of the overlapping genes related to the MAPK signaling pathway in the Venn diagram.

Figure S8

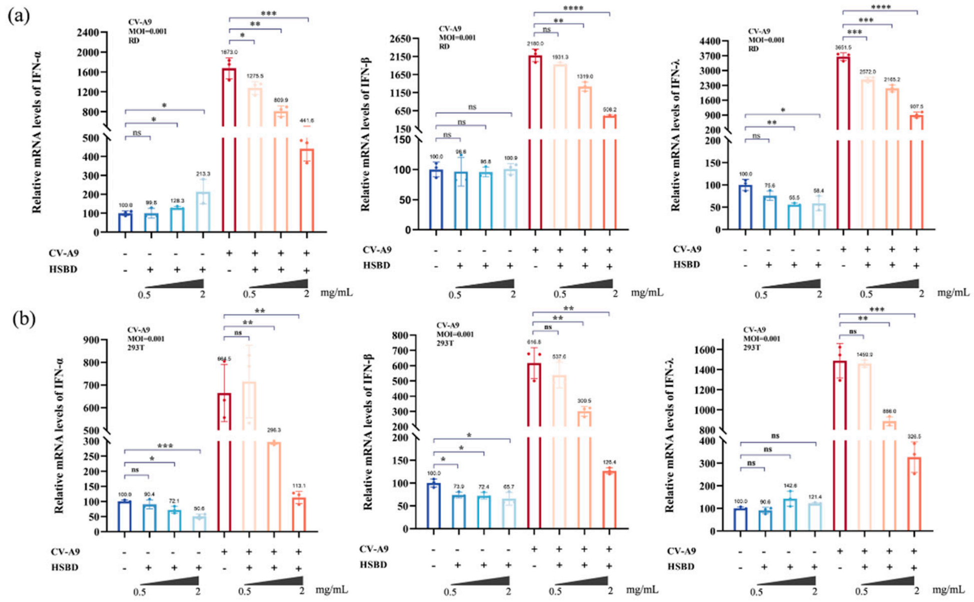

Figure S8. Effect of HSBDF on the expression of IFN-α, IFN-β and IFN-λ induced by CV-A9 infection on RD and 293T cells.

IFN-α, IFN-β and IFN-λ mRNA levels in RD cell **(a)** and 293T cell **(b)** were quantified by RT-qPCR at 24 h.p.i following CV-A9 infection (MOI=0.001) and treatment with 0.5 mg/mL, 1 mg/mL and 2 mg/mL HSBDF. Representative curves of at least two independent experiments and presented as mean ± SD of three technical replicates. ns, no significant difference; ns, no significant difference; * $p < 0.05$; ** $p < 0.01$; *** $p < 0.001$; **** $p < 0.0001$.

Figure S9

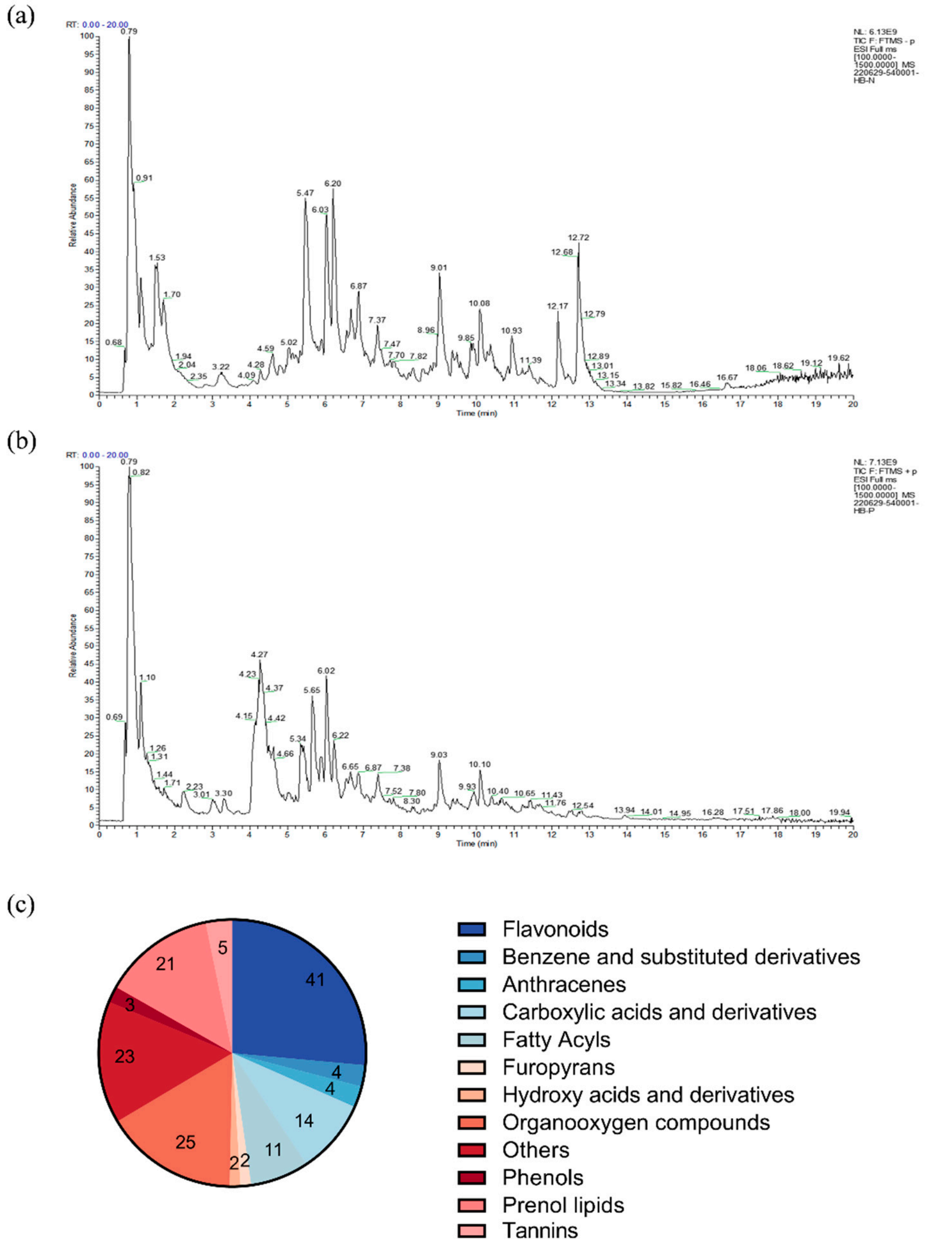

Figure S9. Chemical identification of HSBDF using HPLC-MS/MS.

(a-b) The total ion current (TIC) diagram of the HSBDF in the positive ion mode (a) and negative ion mode (b). **(c)** Statistics of the number of different classes of compounds identified in HSBDF.

Figure S10

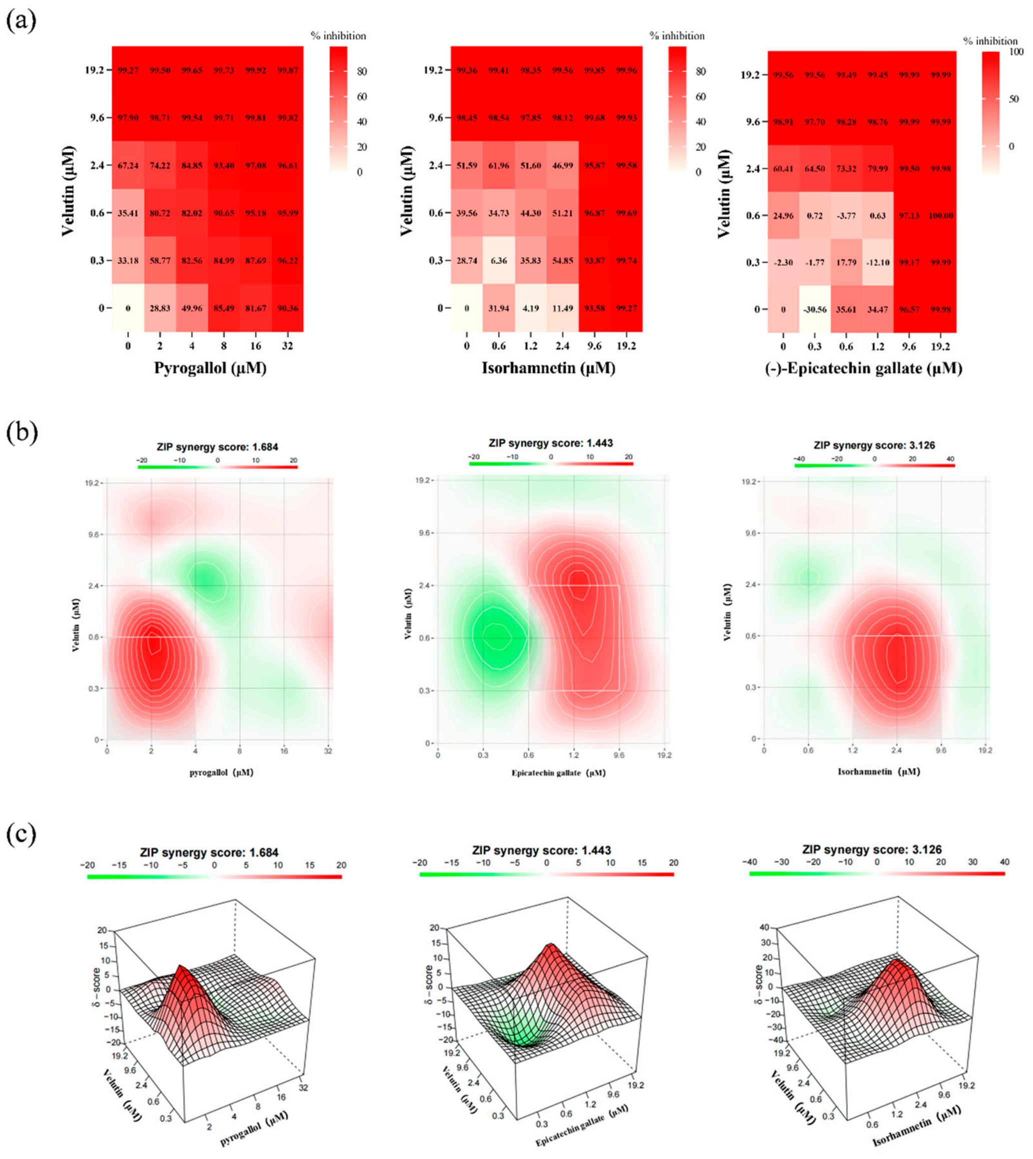

Figure S10. Possibility of the combination of some compounds.

(a) RD cells infected with CV-A9 (MOI=0.001) were co-treated with escalating doses of velutin and pyrogallol (left), isorhamnetin (middle), or (−)-epicatechin gallate (right). Concentration-response matrix of virus-infected cells was analyzed in SynergyFinder, with numbers indicating the percentage of infection inhibition. **(b-c)** Depicting the two-dimensional (b) and three-dimensional (c) topographic maps highlighting areas of synergy across the dose-response matrix. Synergy score: less than -10: the interaction between two drugs may be antagonistic; From -10 to 10: Interactions between two drugs may be additive; Greater than 10: The interaction between two drugs may be synergistic.

Red indicates synergistic action and green indicates antagonistic action between the two drugs.

Figure S11

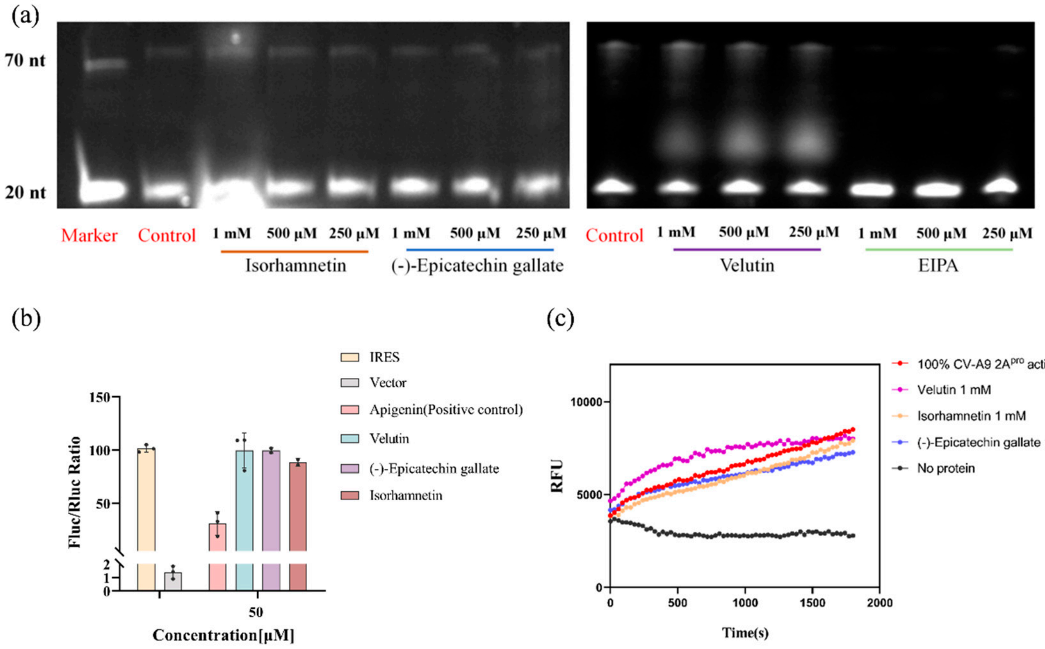

Figure S11. Inhibitory activities of compounds on CV-A9 3D^{pol}, IRES, and 2A^{pro}. **(a)** RNA extension assays were performed to assess the activity of CV-A9 3D^{pol} treated with bioactive compounds, with EIPA as a positive drug. **(b)** The effects of compounds on internal ribosome entry site (IRES) activity were assessed, with the value of Fluc/Rluc as the basis for judging the activity of IRES. Representative curves of at least two independent experiments and presented as mean ± SD of three technical replicates. **(c)** The inhibitory effects of compounds on CV-A9 2A^{pro} were quantified via fluorescence resonance energy transfer (FRET) assay.

Figure S12

(a)

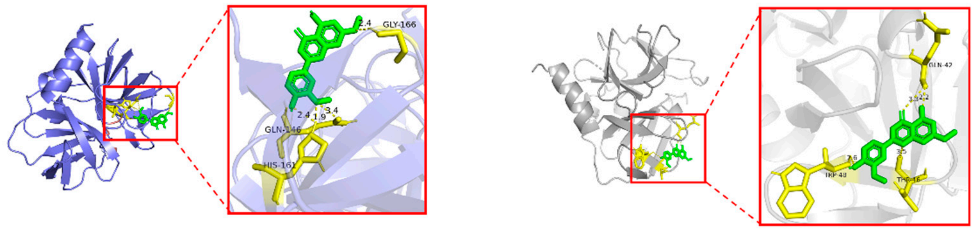

(b)

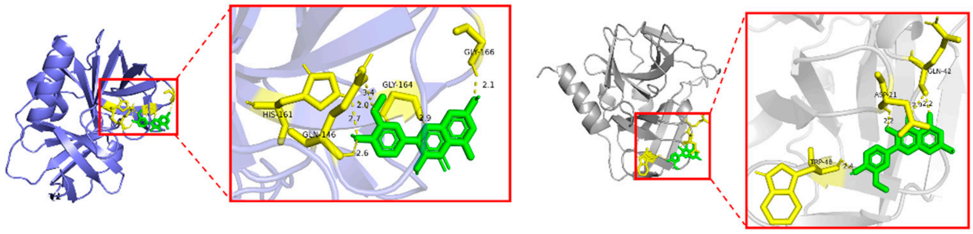

(c)

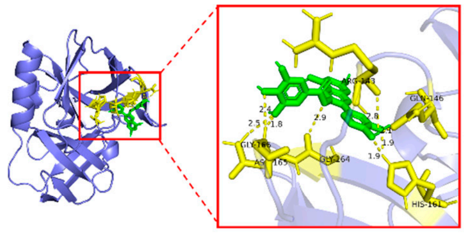

(d)

Protein	Ligand	Binding Energy(ΔG)Kcal/moL	Hydrogen bonding
CVB3 3C protease	Velutin	-5.83	GLN-146、HIS-161、GLY-166
	Isorhamnetin	-4.79	GLN-146、HIS-161、GLY-164、GLY-166
	(-)-Epicatechin gallate	-6.22	ARG-143、GLN-146、HIS-161、GLY-164、ASN-165、GLY-166
EV-A71 3C protease	Velutin	-6.23	GLN-42、THR-46、TRP-48
	Isorhamnetin	-5.05	ASP-21、GLN-42、TRP-48

Figure S12. Docking analysis of compounds with CV-B3 and EV-A71 3C^{pro} models. (a-c) Molecular docking of velutin (a), isorhamnetin (b), and (−)-epicatechin gallate (c) with CV-B3 3C^{pro} (left, blue surface) and EV-A71 3C^{pro} (right, gray surface). Yellow dashed lines denote hydrogen bonds and attached residues. **(d)** Interacting residues and binding energies (ΔG) are tabulated.

Figure S13

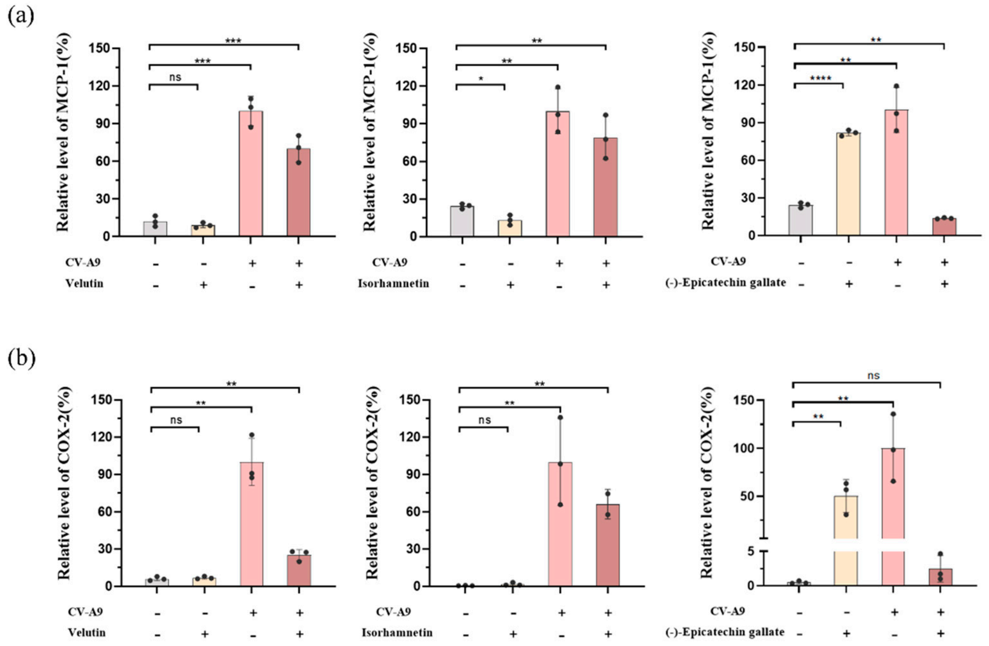

Figure S13. Effect of bioactive compounds on the expression of MCP-1 and COX-2 induced by CV-A9 infection.

MCP-1 **(a)** and COX-2 **(b)** mRNA levels in RD cells were quantified by RT-qPCR at 24 h.p.i following CV-A9 infection (MOI=0.001) and treatment with 50 μM velutin, isorhamnetin, or (−)-epicatechin gallate. Representative curves of at least two independent experiments and presented as mean ± SD of three technical replicates. ns, no significant difference; ns, no significant difference; * $p < 0.05$; ** $p < 0.01$; *** $p < 0.001$; **** $p < 0.0001$.

Table S1. Full spectrum identification results of Huashi Baidu formula (positive ion model). (See in the Excel file)

Table S2. Full spectrum identification results of Huashi Baidu formula (negative ion model). (See in the Excel file)

Table S3. The relevant primer sequences mentioned in the article. (See in the Excel file)

Table S4. Data of the transcriptional difference between virus-treated RD cells and RD cells. (See in the Excel file)

Table S5. Data of the transcriptional difference between HSBD-treated RD cells and virus-treated RD cells. (See in the Excel file)
